# Opioid-free anesthesia with esketamine combined with interpectoral plane block and pectoralis-serratus plane blocks in radical mastectomy: a randomized controlled trial

**DOI:** 10.3389/fphar.2025.1679423

**Published:** 2025-11-25

**Authors:** Wenping Ding, Youming Deng, Nuo Sun, Rongguo Wang, Qian Liu, Yan Zhang, Meiyan Zhou, Yan Liu, Liwei Wang

**Affiliations:** 1 Department of Anesthesiology, Xuzhou Central Hospital, The Xuzhou Clinical College of Xuzhou Medical University, Xuzhou, Jiangsu, China; 2 Department of Anesthesiology, The Second Hospital of Nanjing, Affiliated to Nanjing University of Chinese Medicine, Nanjing, Jiangsu, China; 3 Jiangsu Province Key Laboratory of Anesthesiology, Xuzhou Medical University, Xuzhou, Jiangsu, China

**Keywords:** esketamine, opioid-free anesthesia, interpectoral plane block, pectoralis-serratus plane block, quality of recovery

## Abstract

**Objective:**

Opioid-free anesthesia (OFA) is an emerging technique that aims to reduce or eliminate intraoperative opioid use. Previous studies have shown that OFA is associated with reduced postoperative opioid consumption and fewer adverse events. This study investigated the impact of OFA on the quality of recovery (QoR) in patients undergoing radical mastectomy for breast cancer.

**Methods:**

We enrolled 130 patients scheduled for radical mastectomy and randomly assigned them to either the opioid-free anesthesia (OFA) group or the opioid-based anesthesia (OA) group. Data from 124 patients were ultimately analyzed. The primary outcome was the Quality of Recovery-40 (QoR-40) score at 24 and 48 h postoperatively. Secondary outcomes included the Numerical Rating Scale (NRS) pain scores at 30 min, 24 h, and 48 h postoperatively, perioperative hemodynamic parameters, post-anesthesia care unit (PACU) stay duration, and the incidence of related adverse events.

**Results:**

Postoperative QoR-40 scores were significantly higher in the OFA group than in the OA group at both 24 h (176.1 ± 3.7 vs 169.7 ± 3.3; mean difference 6.4, 95% CI 5.2–7.7, *p* < 0.001) and 48 h (180.3 ± 2.7 vs 173.7 ± 3.6; mean difference 6.6, 95% CI 5.5–7.7, *p* < 0.01). The OFA group showed significantly lower pain scores on the Numeric Rating Scale (NRS) at all measured time points (30 min: 1.89 ± 0.8 vs 2.36 ± 1.2; 24 h: 1.21 ± 0.6 vs 1.66 ± 0.8; 48 h: 1.03 ± 0.4 vs 1.28 ± 0.6, all *p* < 0.05). No statistically significant differences were observed in hemodynamic parameters, including mean arterial pressure (MAP) and heart rate (HR), at the following time points: upon entering the operating room (T0), before anesthesia induction (T1), before tracheal intubation (T2), immediately after tracheal intubation (T3), at skin incision (T4), 10 min after incision (T5), and upon leaving the operating room (T6). However, the incidence of postoperative nausea and vomiting (PONV) differed significantly between the two groups at 30 min, 24 h, and 48 h postoperatively (*p* < 0.05).

**Conclusion:**

For patients undergoing radical mastectomy, opioid-free anesthesia (OFA) utilizing esketamine combined with Interpectoral Plane (IPP) and Pectoralis-Serratus Plane (PSP) blocks significantly improved postoperative recovery quality compared to conventional opioid-based anesthesia (OA).

## 1 Introduction

Breast cancer is one of the most common malignancies in women, and surgery remains the most effective treatment modality. However, postoperative acute and chronic pain represent significant challenges (Altıparmak et al., 2019). Due to the complex innervation of the breast, involving the intercostal (T1-T7), cervical plexus (supraclavicular nerves), and brachial plexus nerves, postoperative pain management is often difficult. Approximately 40% of patients experience severe acute pain after breast cancer surgery, with moderate-to-severe pain persisting in nearly one-third of these cases (Leysen et al., 2017). Consequently, inadequate acute pain management and chronic opioid use are major risk factors for the progression of acute pain into chronic pain. Patients routinely administered opioids after breast cancer surgery exhibit higher pain scores in the immediate postoperative period up to 24 h, along with an increased incidence of persistent postoperative pain. Although opioids effectively alleviate acute pain, their side effects (such as postoperative nausea and vomiting, PONV) and the incidence of chronic postoperative pain remain concerns (Salomé et al., 2021). In recent years, multimodal analgesia (e.g., combined nerve blocks) has been increasingly employed in breast cancer surgery to reduce opioid consumption and related complications.

Currently, opioids remain the most potent and efficacious pharmacological agents for managing various types of pain in clinical practice. They provide effective analgesia and stable intraoperative hemodynamics, making them valuable in the perioperative setting. However, their widespread misuse or excessive use can lead to adverse effects, including hyperalgesia, nausea and vomiting, shivering, and urinary retention (Feenstra et al., 2023). These complications are associated with delayed patient recovery, prolonged stays in post-anesthesia care unit (PACU), delayed discharge, and unplanned hospital readmissions, all of which impose additional burdens on both patients and healthcare resources (Lavand’homme & Steyaert, 2017).

Given the well-documented adverse effects of perioperative opioids, some scholars advocate for minimizing their use. Opioid-free anesthesia (OFA), which employs multimodal non-opioid analgesic techniques to eliminate or reduce intraoperative opioid administration, may serve as a suitable alternative (Massoth et al., 2021). OFA maintains hemodynamic stability and provides antinociceptive effects through multimodal non-opioid agents, such as α-2 adrenergic receptor agonists, local anesthetics, and N-methyl-D-aspartic acid (NMDA)receptor antagonists. In recent years, OFA has demonstrated feasibility and efficacy across various surgical procedures and patient populations. Two meta-analyses revealed that patients subjected to OFA exhibited a lower incidence of PONV compared to those receiving OA, with comparable postoperative pain scores (Da Silveira et al., 2024). Another meta-analysis indicated that OFA significantly reduced postoperative adverse events and opioid consumption (Feenstra et al., 2023). However, some studies report no benefits or inferior outcomes with OFA. A systematic review and meta-analysis of 12 randomized controlled trials (n = 983) showed that OFA reduced postoperative nausea and vomiting (PONV) and short-term analgesic requirements in laparoscopic surgery, but conferred no significant benefit for 24-h pain control or functional recovery (Cheng et al., 2025). Similarly, a study focusing on oral and maxillofacial surgery indicated that although OFA protocols hold some promise, their clinical value remains to be confirmed in larger, well-standardized trials (Qi et al., 2023). Therefore,a key unresolved question is its widespread adoption and optimal clinical indications.

Esketamine, an NMDA receptor antagonist, is used as an anesthetic adjuvant. Subanesthetic doses (0.15–0.3 mg/kg) improve postoperative pain management and reduce opioid requirements while mitigating psychiatric side effects (Shah et al., 2020). Interpectoral Plane (IPP) and Pectoralis-Serratus Plane (PSP) blocks involve the injection of local anesthetics between the pectoralis major and minor muscles and between the pectoralis major and serratus anterior fascia, respectively (Pawa et al., 2018). These blocks target the medial and lateral pectoral nerves, the 2nd to 6th intercostal nerves, and the long thoracic nerve. They are widely used in breast and thoracic surgeries for postoperative analgesia (Versyck et al., 2019).

Therefore, this study hypothesizes that, compared with traditional opioid-based anesthesia, OFA protocol combining esketamine with IPP and PSP blocks will significantly improve postoperative recovery quality in patients undergoing radical mastectomy, and reduce opioid-related adverse effects.

## 2 Materials and methods

### 2.1 Study design

This was a single-center, prospective, randomized, double-blind, controlled trial. In accordance with the CONSORT 2010 guidelines, female patients scheduled for unilateral radical mastectomy at Xuzhou Central Hospital between May 2022 and May 2023 were enrolled. The study protocol was approved by the Ethics Committee of Xuzhou Central Hospital (No. XZXY-LK-20220519–036) and prospectively registered at ClinicalTrials.gov (No. ChiCTR2200061582). A steering committee comprising senior anesthesiologists, surgeons, and statisticians supervised the trial implementation. An independent Data and Safety Monitoring Board (DSMB) conducted quarterly reviews.

### 2.2 Study participants

A total of 130 female patients aged 35–75 years, classified as ASA physical status I-III, with a BMI of 18–30 kg/m^2^, and scheduled for unilateral radical mastectomy were initially enrolled. Due to the impact of pandemic control measures, follow-up data from six participants were lost, leaving 124 subjects for final analysis. Written informed consent was obtained from all participants preoperatively. Exclusion criteria included:Chronic opioid use (>3 months, >10 mg morphine equivalents/day); Contraindications to regional anesthesia (coagulation disorders, infection at the puncture site, local anesthetic allergy); Severe psychiatric disorders (Patient Health Questionnaire-9≥15, Generalized Anxiety Disorder-7≥15); Cognitive impairment (Mini-Mental State Examination <24); Hepatic or renal dysfunction (ALT/AST >3× upper limit of normal, eGFR <60 mL/min/1.73 m^2^).

### 2.3 Randomization and blinding

Randomization was performed by a research nurse using a computer-generated random number sequence (www.random.org) with stratified block randomization (block sizes 4–8) based on anticipated surgical duration (<85 min vs ≥ 85 min). Sequentially numbered, opaque, sealed envelopes were used for allocation concealment. Both patients and surgeons remained blinded to group allocation throughout the study. Healthcare providers responsible for postoperative care and outcome assessments in PACU and ward were also blinded. Due to the necessity of administering general anesthesia, anesthesiologists performing the interventions were not blinded; however, they were excluded from postoperative outcome evaluation and analysis.

### 2.4 Anesthesia and interventions

All patients underwent standard preoperative preparation and education. Upon entering the operating room, baseline vital signs-including electrocardiography (ECG), pulse oximetry (SpO_2_), and body temperature-were routinely monitored. Under local anesthesia, a radial arterial catheter was inserted in the contralateral limb for continuous blood pressure monitoring, and peripheral venous access was secured.

**FIGURE 1:**
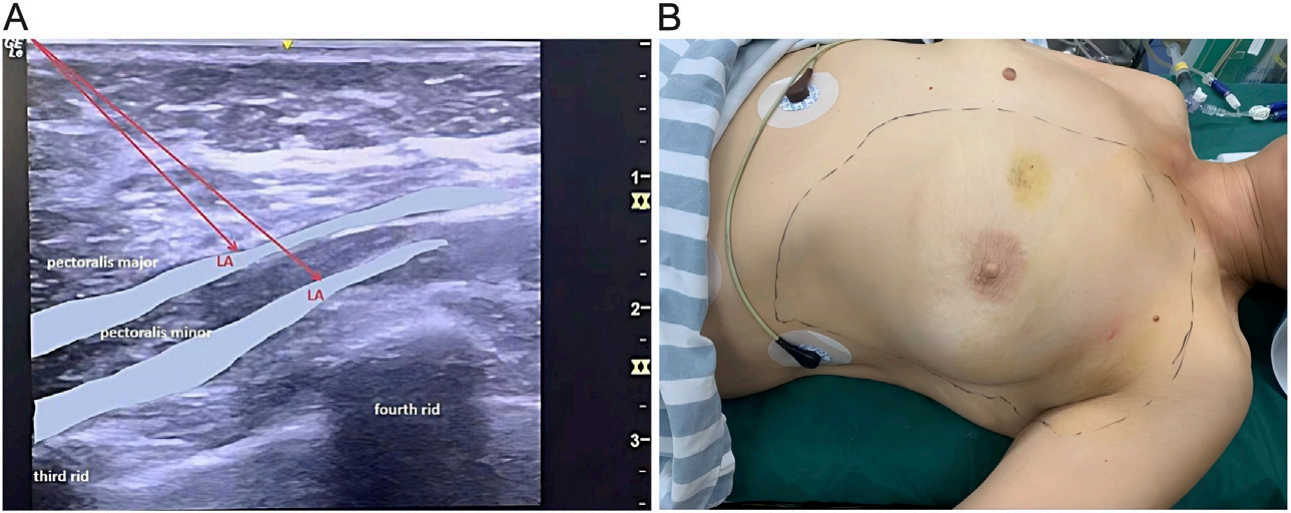
Interpectoral Plane Block and Pectoralis-Serratus Plane blocks. **(A)** Ultrasound-guided injection images for IPP and PSP blocks. **(B)** Sensory block plane evaluation schematic diagram. Sm, Serratus anterior muscle; LA, local anesthetic.

OFA Group: In the pre-induction phase, after monitoring vital signs, ultrasound-guided IPP and PSP blocks were performed using a single-injection technique. The specific procedure was as follows: The patient was placed in the supine position with the affected upper limb abducted. A linear ultrasound probe was positioned at the junction of the middle and lateral thirds of the clavicle. After identifying the axillary artery and vein, the probe was moved distally toward the axilla until the lateral border of the pectoralis minor muscle was visualized. At the level of the 3rd-4th intercostal space, 10 mL of 0.375% ropivacaine was injected between the pectoralis major and minor muscles, followed by 20 mL of 0.375% ropivacaine between the pectoralis minor and serratus anterior muscles. Ten minutes after local anaesthetic injection, pinprick testing was performed in the T2-T6 dermatomes (Figure 1). Loss or clear reduction of sharp sensation was classified as a successful block; absence of such change was deemed block failure. Failed blocks were rescued with ultrasound-guided intercostal nerve block, and the absolute number and proportion of failures were recorded.

During the induction phase, patients received intravenous injections of midazolam (0.05 mg/kg), propofol (2 mg/kg), cisatracurium (0.3 mg/kg), and esketamine (0.3 mg/kg) based on ideal body weight (IBW). The dosage was determined based on the patient’s measured height and weight using the Devine formula. After conirming unconsciousness, absence of eyelash reflex, and no response to verbal stimuli, an appropriately sized endotracheal tube was inserted, and mechanical ventilation was initiated. Intraoperatively, pressure-controlled volume-guaranteed (PCV-VG) ventilation mode was employed with the following parameters: tidal volume 6–8 mL/kg, respiratory rate 12–20 breaths/min, and end-tidal carbon dioxide (EtCO_2_) maintained between 25 and 40 mmHg. Anesthesia was maintained using continuous infusions of propofol (5–10 mg/kg/h) and esketamine (0.3 mg/kg/h). When the propofol infusion rate had already reached 8 mg/kg/h without achieving control,or when sympathetic hyperactivity occurred-defined as heart rate >100 bpm or systolic blood pressure increasing >20% from baseline-sevoflurane at 1%–1.5% was administered. If necessary, additional muscle relaxants were given.

OA Group:For anesthesia induction, patients received midazolam (0.05 mg/kg), propofol (2 mg/kg), cisatracurium (0.3 mg/kg), and sufentanil (0.5 μg/kg) based on ideal body weight. After endotracheal intubation, mechanical ventilation was initiated using the same parameters as described above. Anesthesia was maintained with continuous infusions of propofol (5–10 mg/kg/h) and remifentanil (0.1–0.3 μg/kg/h). If required, consistent with OFA group, 1%–1.5% sevoflurane was administered, or additional muscle relaxants were administered.

From induction until discharge from PACU, hypotension (defined as a systolic blood pressure decrease >20% from baseline and/or systolic blood pressure <90 mmHg) was treated with intravenous norepinephrine (4–8 μg). Bradycardia (defined as a heart rate decrease >20% from baseline and/or heart rate <50 bpm) was treated with intravenous atropine (0.5 mg). Throughout the perioperative period, from PACU stay to post-discharge follow-up, patients reporting NRS pain scores≥4 received an intravenous bolus of 50 mg flurbiprofen axetil. Episodes of vomiting or severe nausea were treated with 10 mg intravenous metoclopramide.

### 2.5 Sample size

The sample size was determined based on the QoR-40 data reported by Myles et al., which indicated a minimal clinically important difference (MCID) of 6.3 (SD = 14) (Myles et al., 2016). Using a two-sided α = 0.05 and β = 0.1, a total of 52 participants were required per group by relevant calculation formula. Considering a 15% dropout rate, the target sample size was set at 122, with 61 participants per group. In this study, 130 patients were initially enrolled; however, due to the impact of pandemic control measures, follow-up data were unavailable for 6 subjects. Consequently, data from 124 participants were included in the final analysis.

### 2.6 Data analysis

Statistical analyses were performed by a researcher blinded to group allocation for all randomized patients. SPSS 24.0 and GraphPad Prism 8.0 were used for statistical computations and graphical representation. The normality of the quantitative variables was tested using the Shapiro–Wilk test. Normally distributed data were expressed as means and standard deviations and analyzed using independent samples t-test. Skewed data were expressed as medians and interquartile ranges and analyzed using MannWhitney U-test. The categorical variables were expressed as numbers (%) and compared using the χ2 or Fisher χ2 test. All tests were two-tailed and statistical significance was considered for *p* < 0.05.

## 3 Results

**FIGURE 2:**
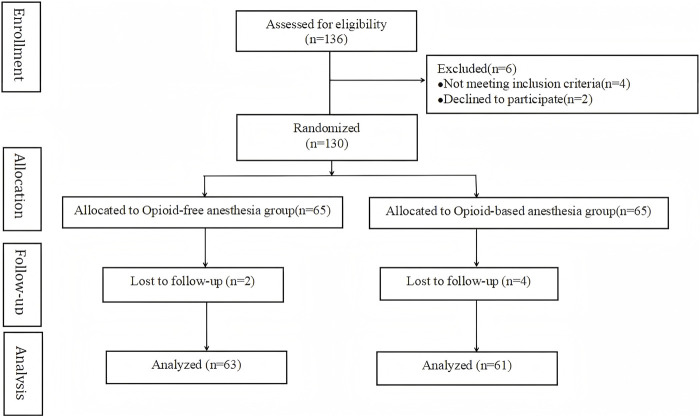
Flow diagram showing the numbers of individuals at each stage of the study.

The detailed enrollment and exclusion flowchart are shown in Figure 2. From May 2022 to May 2023, a total of 136 patients who underwent radical mastectomy for breast cancer at Xuzhou Central Hospital were enrolled in this study. Four failed inclusion criteria, and two declined participation. Hence, 130 patients underwent randomization. Six were excluded due to pandemic-related follow-up issues. Finally, 124 patients (63 OFA, 61 OA) completed the study.

The baseline characteristics of the patients are presented in Table 1. No statistically significant differences were observed in patient baseline characteristics (age,BMI,ASA classification, comorbidities, smoking status) and surgical types (all *p* > 0.05, Table 1).

**TABLE 1:** Patient demographic and perioperative data.

Characteristic	OFA group (n = 63)	OA group (n = 61)	*p*-value
Age (years)	50 ± 9.1	49 ± 9.3	0.669
Height (cm)	160.5 ± 3.4	161.3 ± 3.9	0.252
Weight (kg)	60.2 ± 7.3	61.1 ± 7.5	0.507
BMI (kg/m^2^)	23.3 ± 2.5	23.5 ± 2.6	0.793
ASA physical status			
I	8 (12.7)	8 (13.1)	0.945
II	51 (81)	50 (82)	0.884
III	4 (6.3)	3 (4.9)	0.730
Comorbidities			
Hypertension	14 (22.2)	13 (21.3)	0.902
Diabetes	4 (6.3)	5 (8.2)	0.692
COPD	1 (1.6)	1 (1.6)	0.982
Cardiovascular disease	3 (4.8)	4 (11)	0.665
Current smoker	3 (4.8)	3 (4.9)	0.968
Surgical type			0.959
Modified radical mastectomy	40 (63.5)	39 (63.9)	
Skin-sparing mastectomy	23 (36.5)	22 (36.1)	

Data are mean ± SD, median (IQR), or n (%). *p < 0.05; **p < 0.01; ***p < 0.001. BMI, body mass index. ASA, American Society of Anesthesiologists. COPD, chronic obstructive pulmonary disease.

The total consumption of propofol, midazolam, and cisatracurium during operation showed no statistically significant differences between the two groups (all *p* > 0.05). There were no statistically significant differences between the two groups regarding duration of surgery, time to extubation, length of stay in PACU. However, a significant difference was noted in anesthesia duration between the OFA group and the OA group (*p* < 0.001, Table 2).

**TABLE 2:** Anesthesia management and consumption of anesthetic drugs.

Variables	OFA group (n = 63)	OA group (n = 61)	*p*-value
Propofol (total,mg)	323.6 ± 9.3	321.8 ± 8.4	0.407
Midazolam (mg)	2.42 ± 0.2	2.47 ± 0.2	0.191
Cisatracurium (mg)	12.7 ± 1.0	13.1 ± 1.1	0.061
Esketamine (total,mg)	41.8 ± 2.6		
Sufentanil (ug)		31 ± 2.2	
Remifentanil (ug)		446 ± 56	
Anesthesia time (min)	117.4 ± 10.7	102.4 ± 16.2	<0.001 ***
Surgical time (min)	86.6 ± 12.5	86.8 ± 17.2	0.917
Extubation time (min)	20 (17–26)	18 (16–21)	0.090
PACU stay time (min)	60 (56–68)	61 (56–68)	0.924

Data are mean ± SD, median (IQR), or n (%). *p < 0.05; **p < 0.01; ***p < 0.001. PACU, post-anesthesia care unit.

### 3.1 Primary outcomes

All patients successfully completed the Quality of Recovery-40 (QoR-40) questionnaire. The total QoR-40 score ranges from 40 (poorest recovery) to 200 (optimal recovery), with 6.3 considered the MCID. No significant difference was found in the baseline QoR-40 scores between the two groups (OFA group: 190.8 ± 2.5vs.OA group: 190.9 ± 2.9; mean difference: −0.08, 95% CI [-1.03, 0.9]; *p* = 0.875). However, the primary outcome of this study, the QoR-40 score at 24 h postoperatively, was significantly higher in the OFA group than in the OA group (OFA group 176.1 ± 3.7vs.OA group 169.7 ± 3.3; mean difference 6.4, 95% CI [5.2, 7.7], *p* < 0.001, Table 3). A similar advantage was observed in the QoR-40 score at 48 h postoperatively (OFA group 180.3 ± 2.7vs.OA group 173.7 ± 3.6; mean difference 6.6, 95% CI [5.5, 7.7], *p* < 0.01).

**TABLE 3:** Comparisons of the outcomes and adverse events.

Variables	OFA group (n = 63)	OA group (n = 61)	Difference or RR (95% CI)	*p*-value
Primary outcome				
QoR-40 score preoperative	190.8 ± 2.5	190.9 ± 2.9	−0.08 (-1.03∼0.9)	0.875
QoR-40 score at 24 h	176.1 ± 3.7	169.7 ± 3.3	6.4 (5.2∼7.7)	<0.001 ***
QoR-40 score at 48 h	180.3 ± 2.7	173.7 ± 3.6	6.6 (5.5∼7.7)	<0.01 **
Secondary outcomes				
NRS pain scores				
At 30 min	1.89 ± 0.8	2.36 ± 1.2	−0.5 (-0.8∼-0.1)	0.01 **
At 24 h	1.21 ± 0.6	1.66 ± 0.8	−0.4 (-0.7∼-0.2)	<0.01 **
At 48 h	1.03 ± 0.4	1.28 ± 0.6	−0.2 (-0.4∼-0.06)	0.011 *
Rescue analgesics	1 (1.6)	5 (8.2)	0.2 (0.02∼1.6)	0.086
PONV				
At 30 min	0	7 (11)	0.89 (0.8∼0.97)	0.006 **
At 24 h	3 (5)	18 (30)	0.12 (0.03∼0.43)	<0.001 ***
At 48 h	2 (3.3)	10 (16.7)	0.17 (0.04∼0.8)	0.029 *
Rescue antiemetics	2 (3.3)	11 (18)	0.15 (0.032∼0.7)	0.016*
Shivering	3 (5)	7 (11)	0.25 (0.05∼1.27)	0.151
Pruritus	0	2 (3.3)	0.97 (0.92∼1.01)	0.24
Dizziness or headache	4 (6.3)	9 (14.8)	0.39 (0.11∼1.35)	0.127

Data are mean ± SD, median (IQR), or n (%). *p < 0.05; **p < 0.01; ***p < 0.001. NRS, numerical rating scale. PONV, postoperative nausea and vomiting.

### 3.2 Secondary outcomes

The Numerical Rating Scale (NRS) scores for pain at 30 min, 24 h, and 48 h postoperatively were significantly lower in the OFA group than in the OA group (30 min: OFA group 1.89 ± 0.8vs.OA group 2.36 ± 1.2; 24 h: OFA group 1.21 ± 0.6 vs. OA group 1.66 ± 0.8; 48 h: OFA group 1.03 ± 0.4 vs.OA group 1.28 ± 0.6) (Table 3). However,the rescue analgesic administration rates did not differ significantly between groups (OFA: 1.6% vs. OA: 8.2%; *p* > 0.05).

**FIGURE 3:**
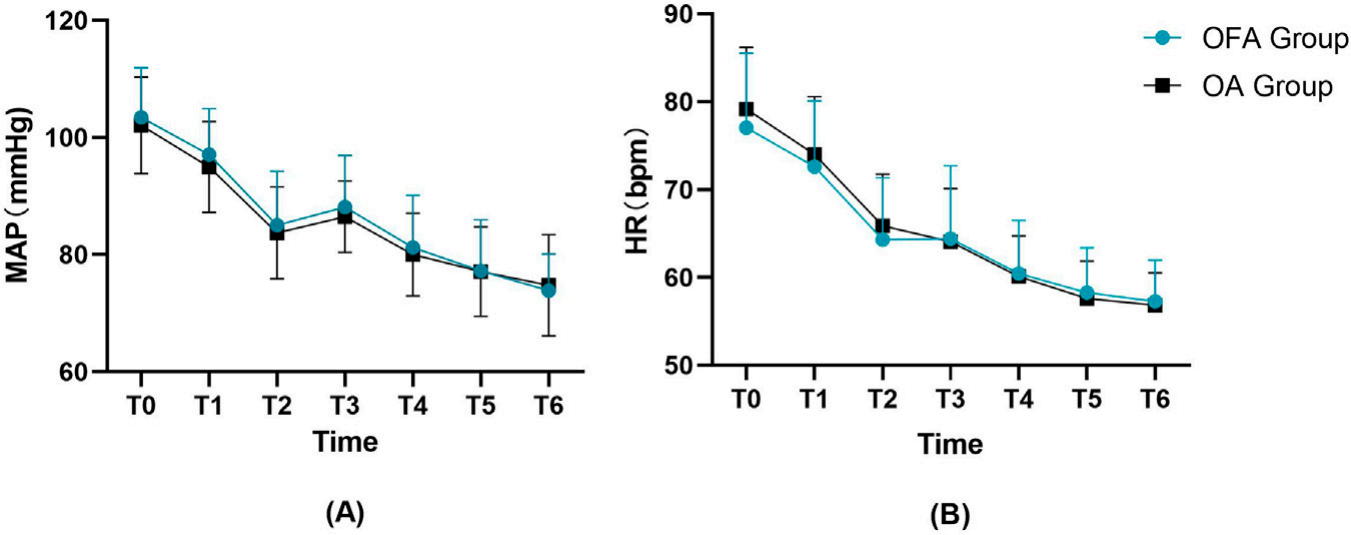
Perioperative hemodynamic variables including **(A)** MAP, **(B)** HR at seven time points. HR, heart rate; MBP, mean blood pressure. T0,upon entering the operating room. T1,before anesthesia induction. T2,before tracheal intubation. T3,immediately after tracheal intubation. T4,at skin incision. T5,10 min after skin incision. T6, upon leaving the operating room.

Additionally, perioperative hemodynamic parameters, including mean arterial pressure (MAP) and heart rate (HR), were analyzed at the following seven time points: upon entering the operating room (T0), before anesthesia induction (T1), before tracheal intubation (T2), immediately after tracheal intubation (T3), at skin incision (T4), 10 min after skin incision (T5), and upon leaving the operating room (T6). As shown in Figure 3, no statistically significant differences were observed in MAP or HR between the two groups at any time point (all *p* > 0.05).

### 3.3 Adverse events

Significant differences were observed in the incidence of PONV between the two groups at 30 min (OFA group: 0% vs. OA group: 11%; *p* < 0.01), 24 h (OFA group: 5% vs. OA group: 30%; *p* < 0.001), and 48 h (OFA group: 3.3% vs. OA group: 16.7%; *p* < 0.05) (Table 3). Furthermore, the rates of rescue antiemetic administration also differed significantly between the two groups. However, no significant differences were observed in other adverse events (such as shivering, pruritus, dizziness, headache) occurring from the end of surgery to 48 h postoperatively.

## 4 Discussion

This single-center, randomized, double-blind controlled trial aimed to evaluate the effects of OFA with esketamine in combination with IPP and PSP blocks on the quality of recovery in patients undergoing radical mastectomy. The results revealed significantly enhanced quality of recovery in the OFA group compared to OA group. Importantly, intraoperative hemodynamic stability was well-maintained in both groups, with comparable anesthetic indices, demonstrating the safety and feasibility of the OFA approach for this surgical procedure while providing effective analgesia. Furthermore, postoperative assessments showed that the OFA group exhibited consistently superior outcomes, including: (1) significantly lower pain scores at all measured time points (PACU, 24h, and 48 h postoperatively); (2) a markedly reduced incidence of PONV; and (3) fewer episodes of shivering compared to the OA group.

It is noteworthy that our findings appear to contrast with a growing body of literature reporting neutral or even unfavorable outcomes for OFA. Specifically, a recent systematic review and network meta-analysis of 42 RCTs demonstrated no statistically significant difference in 24-h postoperative pain intensity between OFA and OA regimens (Tripodi, 2025). Similarly, another meta-analysis of 983 patients (Cheng et al., 2025) confirmed that while OFA effectively reduced PONV incidence and early analgesic demand in laparoscopic surgery, these benefits did not translate to superior pain control or enhanced functional recovery in the postoperative period. A randomized trial by Brescia et al. (2025) found that opioid-free general anesthesia combined with thoracic-wall blocks did not improve chronic pain outcomes up to 24 months after quadrantectomy for breast cancer. Admittedly, the absence of long-term follow-up in our study means we cannot assess this particular endpoint. However, the use of a dual-plane (IPP-PSP) block, which covers the T2-T6 dermatomes, could theoretically provide a more complete afferent nociceptive blockade than a single-plane technique. We hypothesize that this extensive blockade may mitigate the transition from acute to persistent pain-a premise that must be validated in future, adequately powered long-term trials. Collectively, while OFA demonstrates potential in reducing PONV and opioid consumption, the available evidence is marked by considerable heterogeneity and generally low-to-moderate quality. Therefore, well-designed, multicenter RCTs with standardized protocols are urgently needed to conclusively determine its clinical value.

This study demonstrates that OFA achieves significantly higher QoR-40 scores compared to OA in radical mastectomy patients (176.1 ± 3.7 vs 169.7 ± 3.3 at 24 h; *p* < 0.001) The QoR-40 scale is a validated tool for assessing postoperative recovery quality and has been widely applied in various clinical settings (Gornall et al., 2013). According to prior research by Dr. Myles, it encompasses five dimensions: emotional state, physical comfort, psychological support, physical independence, and pain. The total score is the sum of all item scores, ranging from 40 to 200, with higher scores reflecting better recovery experiences, emphasizing subjective wellbeing and patient satisfaction postoperatively (Myles et al., 2000). Chen et al. adapted and validated the Chinese version of this scale, confirming its reliability and feasibility for evaluating postoperative recovery in Chinese patients (Chen et al., 2020). They demonstrated that the QoR-40 questionnaire can be independently completed within approximately 7 min, demonstrating excellent clinical feasibility. Furthermore, the QoR-40 scale exhibits acceptable reliability, validity, and responsiveness, making it suitable for assessing health recovery at different postoperative time points in Chinese surgical patients.

Regarding clinical interpretation, the minimal clinically important difference (MCID) - defined as the smallest score change perceived as beneficial by patients - was established as 6.3 points for the QoR-40 scale by Myles et al. In our study, the OFA group achieved clinically meaningful improvements over OA, with between-group differences exceeding this MCID threshold at both 24 h (mean difference 6.4, 95% CI [5.2–7.7]) and 48 h (mean difference 6.6, 95% CI [5.5–7.7]) postoperatively. These results not only demonstrate statistical significance but also confirm the clinical relevance of OFA in enhancing postoperative recovery.

Esketamine, the S-enantiomer of racemic ketamine, is a potent NMDA receptor antagonist with demonstrated analgesic efficacy in perioperative settings. As an adjunct to general anesthesia, subanesthetic doses of esketamine provide effective analgesia while maintaining a favorable safety profile (Zhu et al., 2022). Clinical evidence indicates that low-dose esketamine (0.15–0.5 mg/kg) significantly reduces postoperative opioid requirements and mitigates opioid-related adverse effects, including nausea and vomiting, thereby serving as a valuable component of multimodal analgesia strategies. While higher doses (e.g., 0.5 mg/kg) may offer enhanced analgesic benefits, their clinical utility is limited by dose-dependent adverse effects, such as transient hypertension and psychomimetic symptoms (Li et al., 2025). Furthermore, pharmacodynamic studies have established the median effective dose (ED50) and 95% effective dose (ED95) of esketamine for early postoperative pain control at 0.301 mg/kg and 0.379 mg/kg, respectively (Xu et al., 2023). Based on these pharmacokinetic parameters, this study employed a dose of 0.3 mg/kg for both induction and maintenance, achieving an optimal therapeutic window that balances analgesic efficacy with safety considerations. This dosing regimen aligns with previous clinical evidence demonstrating that intraoperative esketamine administration (0.25 mg/kg bolus followed by 0.125 mg/kg/h infusion) significantly enhances postoperative recovery, as evidenced by a 7-point improvement in QoR-40 scores at 48 h, reduced pain scores, and decreased requirement for rescue analgesia (Cheng et al., 2022). Notably, this pharmacological approach may be particularly beneficial for breast cancer patients, among whom 20%–45% experience postoperative anxiety and depression, conditions that may be ameliorated by esketamine’s unique psychotropic properties (Movahed et al., 2025).

Esketamine demonstrates a unique multimodal pharmacological profile, combining sedative-analgesic properties with rapid-onset anxiolytic and antidepressant effects when administered intravenously. Clinical evidence from a recent randomized trial confirms that a single subanesthetic dose (0.3 mg/kg) administered post-induction significantly reduces both postoperative pain and anxiety in surgical patients (Gong, 2023). The underlying mechanisms likely involve dual pathways: NMDA receptor antagonism leading to subsequent 5-HT level elevation, and neuroplasticity induction that counteracts stress- and depression-induced neuronal damage. These neurobiological effects collectively contribute to esketamine’s distinctive psychotropic benefits (Hu et al., 2025). Regarding postoperative recovery, our findings corroborate existing literature showing significantly higher PONV incidence in OA versus OFA groups following radical mastectomy. This difference may be attributed to two principal factors: first, the inherent emetogenic properties of intraoperative opioids; and second, their secondary effects on sleep architecture and nutritional intake. As an effective opioid-sparing agent, esketamine not only provides comparable analgesia but also substantially reduces these opioid-related complications, particularly nausea and vomiting.

The IPP and PSP blocks demonstrate broad clinical applicability for anterior thoracic wall procedures, including radical/modified radical mastectomy, axillary lymph node dissection, breast reconstruction, and sternotomy. These techniques provide comprehensive regional anesthesia encompassing both breast and axillary territories through a dual-injection approach (Genc et al., 2022). The first injection delivers local anesthetic into the fascial plane between the pectoralis major and minor muscles, effectively targeting both the medial (C8-T1) and lateral (C5-C7) pectoral nerves that provide motor innervation to the pectoralis major. The subsequent injection is performed more laterally in the plane between the pectoralis minor and serratus anterior muscles, achieving:sensory blockade of the lateral cutaneous branches from T2-T6 intercostal nerves, anesthesia of the thoracic wall (chest and axillary regions),and motor blockade of the long thoracic nerve (C5-C7) innervating the serratus anterior. This anatomical approach ensures complete coverage of the surgical field while minimizing systemic analgesic requirements. (Senapathi et al., 2019).

The efficacy of IPP and PSP blocks has been demonstrated in published RCTs and retrospective studies (Genc et al., 2022; Morioka et al., 2015; Versyck et al., 2019). Research on modified radical mastectomy revealed that the combined IPP-PSP block technique significantly reduces both intraoperative opioid consumption and postoperative analgesic requirements compared to conventional approaches, with concomitant improvements in pain scores as measured by Visual Analog Scale (VAS) (Shah et al., 2020). Building upon this evidence, our study employs the integrated IPP-PSP blockade strategy to optimize perioperative pain management and enhance recovery quality in radical mastectomy patients. From a hemodynamic standpoint, the OFA protocol incorporating esketamine with IPP-PSP blocks provides stable anesthetic conditions throughout the surgical procedure, maintaining adequate sedation and analgesia while minimizing cardiovascular instability. These findings substantiate that the synergistic combination of esketamine and regional anesthesia techniques (IPP-PSP) can effectively serve as an opioid-sparing alternative, corroborating previous research on multimodal analgesic approaches in oncologic breast surgery (Elshanbary et al., 2021).

Furthermore, our study demonstrated significant between-group differences in the reduction of opioid-related adverse symptoms (ORAS), particularly for nausea/vomiting and analgesic requirements. Importantly, these improvements directly correlated with enhanced QoR scores. This synergy-reducing ORAS while improving recovery metrics-is well aligned with the core enhanced recovery after surgery (ERAS) objectives of minimizing surgical stress and expediting rehabilitation. Consequently, we propose this OFA regimen as a viable and valuable component of the anesthetic protocol for radical mastectomy within an ERAS pathway. These findings resonate with contemporary evidence, which confirms that multimodal OFA strategies can effectively mitigate surgical stress responses, optimize perioperative care pathways, and ultimately preserve postoperative functional status and quality of life (Chen et al., 2023). Notably, the incidence of other ORAS (shivering, pruritus, and dizziness/headache) showed no significant intergroup differences, which may reflect the relatively short operative duration characteristic of radical mastectomy procedures. These results suggest the need for further investigation in more complex surgical scenarios to comprehensively evaluate the differential effects of these anesthetic approaches on ORAS incidence.

For conclusion,the superior recovery quality in the OFA group may be attributed to the multimodal pharmacological effects of esketamine, including NMDA receptor antagonism, opioid receptor modulation, and anti-inflammatory properties, synergizing with the sensory blockade provided by IPP and PSP techniques. Unlike conventional polypharmacy OFA regimens utilizing dexmedetomidine, lidocaine, magnesium, or clonidine, this study establishes the clinical efficacy of a novel esketamine-based OFA protocol combined with dual plane blocks (Di Benedetto et al., 2021; Xue et al., 2024). Our results demonstrate strong concordance with the seminal work by Yu et al., wherein esketamine combined with pectoral nerve blocks significantly enhanced postoperative recovery, as evidenced by improved QoR-40 scores at discharge and reduced anxiety/depression scores in modified radical mastectomy patients (Yu et al., 2022). Importantly, their findings of attenuated inflammatory responses (decreased IL-6 levels at 24 h postoperatively) and superior patient satisfaction scores further validate the clinical efficacy of this anesthetic approach - observations that are strongly supported by our current dataset. These conclusions are further reinforced by two recent high-quality meta-analyses, which collectively demonstrate that OFA protocols: (1) significantly improve early postoperative recovery quality (QoR-40 scores within 24 h), (2) reduce both pain scores and analgesic requirements, and (3) substantially decrease the risk of PONV compared to conventional opioid-based regimens (Liu et al., 2025; Wang et al., 2025).

The OFA regimen implemented in this study successfully reduced opioid consumption and the incidence of PONV while enhancing postoperative analgesia, without compromising surgical conditions. However, this study has several important limitations that should be acknowledged. First, the observational period was restricted to 48 h postoperatively, precluding assessment of long-term recovery outcomes and potential chronic pain development. Therefore, this study cannot determine the impact of OFA on long-term patient prognosis. Second, while we utilized the comprehensive QoR-40 scale, we did not perform subgroup analyses of its five constituent domains (emotional state, physical comfort, psychological support, physical independence, and pain), which could have provided more nuanced insights into recovery patterns. Third, our OFA protocol focused specifically on esketamine-based analgesia and did not compare alternative multimodal approaches incorporating agents such as lidocaine or dexmedetomidine. Finally, as a single-center trial with a modest sample size (n = 124), our findings may be subject to selection bias and require validation through larger, multicenter investigations to ensure generalizability across diverse clinical settings.

## 5 Conclusion

In summary, this randomized controlled trial demonstrates that OFA utilizing esketamine in combination with IPP and PSP blocks significantly enhances postoperative recovery quality at both 24 and 48 h timepoints compared to conventional opioid-based anesthesia in radical mastectomy patients. These findings position OFA as a clinically viable anesthetic strategy that not only improves recovery metrics but also effectively mitigates opioid-associated adverse effects, offering a promising alternative within ERAS protocols for breast cancer procedures.

## Data Availability

The original contributions presented in the study are included in the article/Supplementary Material, further inquiries can be directed to the corresponding authors.
