# Supplementary material for: Opioid-free anesthesia with esketamine combined with interpectoral plane block and pectoralis-serratus plane blocks in radical mastectomy: a randomized controlled trial
Source: Front Pharmacol. 2025 Nov 25;16:1679423. doi: 10.3389/fphar.2025.1679423 (PMC12746497; doi:10.3389/fphar.2025.1679423)
Supplement: Supplementary file 1 [file Table1.docx]

**Supplementary TABLE 1 Patient hemodynamic data at different time points.**

|  | OFA Group (n=63) | OA Group(n=61) | *p*-value |
| --- | --- | --- | --- |
| MAP |  |  |  |
| T0 | 103±8.5 | 102±8.3 | 0.368 |
| T1 | 97±7.8 | 95±7.7 | 0.137 |
| T2 | 85±9.2 | 84±7.9 | 0.396 |
| T3 | 88±8.8 | 86±6.1 | 0.234 |
| T4 | 81±8.9 | 80±7.1 | 0.419 |
| T5 | 77±8.8 | 77±7.6 | 0.951 |
| T6 | 74±6.3 | 75±8.7 | 0.509 |
| HR |  |  |  |
| T0 | 77±8.4 | 79±7.1 | 0.132 |
| T1 | 73±7.5 | 74±6.7 | 0.278 |
| T2 | 64±7.0 | 66±5.9 | 0.189 |
| T3 | 64±8.3 | 64±6.0 | 0.791 |
| T4 | 60±6.1 | 60±4.7 | 0.710 |
| T5 | 58±5.1 | 58±4.2 | 0.433 |
| T6 | 57±4.7 | 56±3.7 | 0.554 |
